# Self-assembly of Deinococcus radiodurans supports nanocell scenario of life origin

**DOI:** 10.15190/d.2017.2

**Published:** 2017-05-08

**Authors:** Alexandra Bernadotte, Valeriya Semenova, Vitor A. M. Musial, Anna Kasprzykowska, Roman A. Zubarev

**Affiliations:** Division of Physiological Chemistry I, Department of Medical Biochemistry and Biophysics, Karolinska Institutet, Stockholm, Sweden; Faculty of Mechanics and Mathematics, Lomonosov Moscow State University, Moscow, 119991, Russian Federation; SciLifeLab, Stockholm, Sweden

**Keywords:** Bacterial self-assembly, nanocell, life origin

## Abstract

In many origin-of-life scenarios, first a kit of elements and simple compounds emerges, then a primitive membrane and then a nanocell with a minimal genome is self-assembled, which then proceeds to multiply by copying itself while mutating. Testing this scenario, we selected Deinococcus Radiodurans known for its exceptional self-repair properties as a model system, separated its bacterial lysis into DNA, RNA and protein fractions, while lipids were used for liposome formation. The fractions were sealed in glass tubes individually and in combinations and stored for three weeks. Upon seeding on Petri dishes, the fractions containing liposomes together with nucleic acid and/or proteins gave in total 19 colonies of Deinococcus radiodurans (confirmed by proteomics), while liposome-free fractions as well as liposome-only fractions gave no colonies. The self-assembly of viable cells from essentially dead mixtures validates the lyposome-based origin-of-life scenario.

## INTRODUCTION

How did Life emerge on this planet? Scientists are yet to agree on a single convincing answer. Half a century later after Darwin's “warm little pond” (1871), Alexander Oparin formulated the "Primordial soup" hypothesis (1924), which suggested that complex organic molecules can form abiogenically from simple molecules under the reducing conditions of early Earth. The hypothesis was corroborated by Miller-Urey experiments^1,2^.Recently, our group has shown that Miller-Urey mixture can support life of bacteria^3^. But the big issue remains unsolved: even given all the right ingredients, how can a living organism form from dead matter?

One possible answer is self-organization. The classical example is the ribosome, the cellular machine that builds proteins. It has more than 50 different parts (proteins and RNAs), and it can assemble itself from them when they are available in reasonable concentrations. Another example is bacterial micro-compartments, which are large organelles formed by the self-assembly of hundreds of proteins into one well-defined structure^4,5^. But there is still a large gap between self-assembly of a biological compartment and a living cell.

How can a complex conglomerate of cellular machinery become a viable cell? In some origin-of-life scenarios, this gap is filled by the so-called nanocell, a minimum-sized structure formed by self-assembling lipid membrane and containing a minimal genome^6^. Once self-assembled, the nanocell then proceeds to multiply by copying itself while mutating. The advantageous mutations associated with multiple copies of the ribosome are accumulated, and the nanocell keeps growing, while increasing the rate of multiplication, until a viable full-size cell evolves.

The above scenario is plausible, but in experimental testing it has never produced a living cell. It is likely that the success of the experiment depends critically upon the choice of a model organism. Here we design such as test using a bacterium that possesses strongest known self-repairing and self-assembling properties. *Deinococcus radiodurans *is known for its ability to survive extreme conditions, such as cold, dehydration, high vacuum, radiation and acid^7^. *Deinococcus radiodurans* is best of all known bacteria able to resist the cytotoxic and genotoxic effects of ionizing radiation, ultraviolet (UV) light, oxidizing agents such as hydrogen peroxide, and other DNA damaging chemicals. This special repair ability *Deinococcus radiodurans *owes to its unique and incredibly effective protein-based mechanism of double-strand break repair^8^. To survive in extreme conditions,* Deinococcus radiodurans* learned to fix not just broken DNA, but also damaged membranes, organelles and other structural cellular components. Moreover, *Deinococcus radiodurans *can build in its DNA into a recipient genome, such as another *Deinococcus,* or even a different species, e.g., *H. influenzae^9^*. All these extreme repair and survival properties make *Deinococcus radiodurans *an optimal candidate for the self-assembly experiment. The main hypothesis was that the cytoplasm content of simple prokaryote organisms supplemented with simple liposomes derived from the lipids composing bacterial cell walls could give rise to a viable self-replicating cell bearing resemblance, or even identical, to the original bacterium.

**Figure 1:**
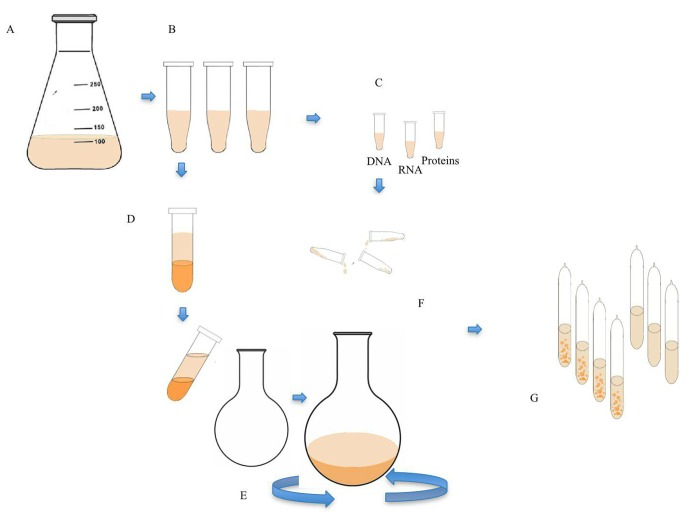
Sample preparation - experimental outline **A.***Deinococcus Radiodurans* growth. **B.** Centrifugation. **C.** Isolation of RNA, DNA and proteins. **D.** Lipid isolation. **E.** Lipid dehydration by rotary evaporation to form dry thin lipid film. **F.** Liposome preparation and loading with combinations of RNA, DNA, and proteins suspended in M9 media. **G.** Sealing of the prepared samples into glass vials and their storage.

We performed two independent experiments, one during the summer of 2016 and another one during the fall of the same year, with a two-months interval. The experiments consisted of growing a large volume of *Deinococcus radiodurans *bacteria, destroying them by separating lipids, DNAs and RNAs (**Table 1**), and then reassembling these parts into one mixture and incubating it in favorable conditions for a period of time (**Figure 1**). To test whether even a single living cell emerged from this mixture through self-assembly, the incubated solution was seeded onto growth media, and the emerging colonies, if any, were counted. To verify that the growing organisms were indeed *Deinococcus radiodurans*, proteomics analysis was performed on the bacteria from the colonies, and the results compared to the proteome of the original *Deinococcus *bacteria. For positive control, living bacteria were sealed; for negative control, pure media or liposome-free biopolymers were encapsulated.

**Table 1:** Composition of test and control solutions of hydrating medium

Components	Test 1 RNA	Test 2 DNA	Test 3 proteins	Test 4 RNA+DNA +proteins	Negative control solution	Negative control solution	Positive control solution
M9 minimal Medium	1 ml	1 ml	1 ml	1 ml	1 ml	1 ml	1 ml
RNA of D. Radiodurans	1 mg/ml			1 mg/ml		1 mg/ml	1 mg/ml
DNA of D. Radiodurans		1 mg/ml		1 mg/ml		1 mg/ml	1 mg/ml
Proteins of D. Radiodurans			1 mg/ml	1 mg/ml		1 mg/ml	1 mg/ml
D. Radiodurans							1 mg/ml
Liposomes	+	+	+	+	+	+	+

## MATERIALS AND METHODS

### Microbial Growth

A large population of *Deinococcus Radiodurans* was obtained by growing for 5 days in 250 mL flasks (of the total volume of 10 L) at 25 ^o^C in minimal M9 media seeded to OD=0.2. Harvesting of bacteria was performed at the exponential growth phase when OD reached 1.2, which corresponds to ca. 10^9^ *Deinococcus Radiodurans* bacteria per mL. Media with bacteria were then centrifuged in 15 mL plastic tubes at 10,000 g for 10 min.

### RNA, DNA, and protein isolation

RNA, DNA and proteins were isolated using Norgen’s RNA/DNA/Protein Purification Kit according to manufacturer’s protocol (Supplementary Figure 1). In short, lysozyme-containing RNAse-free TE Buffer was added directly to the frozen bacterial pellet with ≈10^9^ bacteria. The lysate was applied onto the RNA and DNA purification columns (600 μL per column) and centrifuged at 5,000 x g for 1 min. The flow-through was collected, transferred to the Proteins Activated Column, with pH adjusted using Protein pH Binding Buffer. The proteins were washed and eluted from the column, using respective solutions from the kit. The RNA and DNA Purification Columns were then washed and nucleic acids eluted, first RNAs and then DNAs, with the respective kit buffers.

### Isolation of Lipids

Lipid isolation was based on the well know Folch **method^10,11^; as a protocol modification, NaCl was not used on the final stage of isolation. The medium with bacteria was centrifuged at 12,000 g for 10 min at 4 °C. The obtained pellets were re-suspended in chloroform/methanol (2:1) solution in proportion 1:20 for 20 min at room temperature (RT) in an orbital shaker. The suspension was centrifuged at 2,000 g for 10 min, and the liquid phase was collected and vacuum-evaporated on a Speedvac. To verify the success of lipid extraction, 1% osmic acid was used (the color turns black in presence of lipids).

### Liposome preparation by hydration

To prepare large (500-600 nm) lipid vesicles, which are still small compared to *Deinococcus Radiodurans* diameter of 1500-3500 nm^12^, we used the reverse-phase evaporation^13-15^, as well as DNA-loading of liposomes^16^.

In brief, 800 mg of lipids extracted by modified Folch method were dissolved in 10 mL of chloroform/ethanol (2:1) in a 25 mL round bottom flask^17^. The solvent was then removed by rotary evaporation at 40 °C to obtain a dry thin lipid film on the inside surface of the flask. The liposome preparation was performed by addition of hydrating medium solutions (**Table 1**).

To each flask with dry lipid film, 3 mL of prepared solution were added. The lipid suspension was maintained slightly above the temperature for gel - liquid crystal transition in lipids (≈40°C for *Deinococcus Radiodurans* lipids, according to our observations) during the hydration period of 2 h with vigorous shaking in a sterile hood.

### Confinement in an isolated system 

Solutions (3 mL) were injected into sterile glass tubes with 0.8 mm ID and 8 cm long. Each type of solution was injected into 3 glass tubes (replicates). The glass tubes were sealed using the FireBoy glass-welding device, shaken at 300 rmp for 3 h at RT, and stored at +4 °C (Supplementary Figure 2), with 1 h of shaking at RT every day.

### Verifying bacterial viability 

After 3 weeks of storage, the glass tubes were opened in sterile environment and seeded by streaking onto freshly prepared TGY-Agar Petri dishes (TGY: 5 g Pancreatic digest of casein (Tryptone, Sigma-Aldrich), 5 g Yeast Extract (Yeast Extract for microbiology, Sigma-Aldrich), 1 g glucose, 1 g K_2_HPO_4_, and 7.5 g agar per 1 L of H_2_O). Three Petri dishes were prepared for each glass tube. The dishes were incubated at 25 °C for 10 days. The colonies that appeared on the surface were counted and transferred to sterile 15 mL plastic tubes with fresh TGY medium and incubated at 37 °C while shaking until OD exceeded 0.2. After that the medium was centrifuged at 5,000 rpm for 10 min at 10 °C and the pellets were collected and stored at -20 °C for further analysis.

### Protein extraction 

Bacterial pellets were dissolved in 200 μL of lysis buffer (AmBic 50 mM, SDC 3% w/v, 1 tablet of protease inhibitors x10 mL) and kept on ice for 10 min. The solution was then sonicated using the pulse mode for 2 min, centrifuged at 12,000 g for 10 min at 2 °C and supernatant was collected. BCA assay was used to check the protein concentration. 100 µg of protein were precipitated in acetone at -20 °C, with 60 min incubation and 10 min centrifuging at 14,000 g. Proteins were dried in a SpeedVac and resuspended in 100 µL of 8 M urea and 50 mM Tris buffer with pH 8.5. Protein disulfide bond reduction was performed by 1 h incubation with DTT added to a final concentration of 5 mM. Alkylation of the reduced cysteines was done by 1 h incubation in the dark with iodoacetamide added to a 50 mM final concentration. The latter reaction was quenched by adding 10 mM DTT, after which the solution was diluted with the same volume of 50 mM Tris at pH 8.5. Proteins were digested first with LysC (1:100 molar ratio) at RT overnight. The trypsin digestion was then performed by 6 h incubation at RT with 1 µg of sequencing-grade trypsin added per 100 µg of proteins with 3 volumes of 50 mM Tris at pH 8.5. The obtained peptides were desalted by binding to SepPak columns, washing with 0.1% TFA water solution followed by elution with 50% ACN.

### Proteome analysis

Proteome analysis was performed by LC-MS/MS with a 2 h LC gradient on an LTQ Orbitrap Elite mass spectrometer (Thermo Scientific), using the resolution of 100,000 and the highest target value for MS (survey mass spectra), and the resolution of 30,000 for HCD MS/MS (fragmentation mass spectra). The peptides and proteins were searched by Mascot (Matrix Science, UK) in a database (UniProt v. 2.5, 3119 sequences of* Deinococcus radiodurans*) using the following parameters: mass tolerance of 5 ppm for MS and 20 mDa for MS/MS, 1% false discovery rate. Quantification was performed with Quanti software v. 2.5.4.4 using the label-free approach^18^ and at least 2 unique peptides per protein.

## **R**ESULTS

### Bacterial colonies on Petri dishes

**Figure 2:**
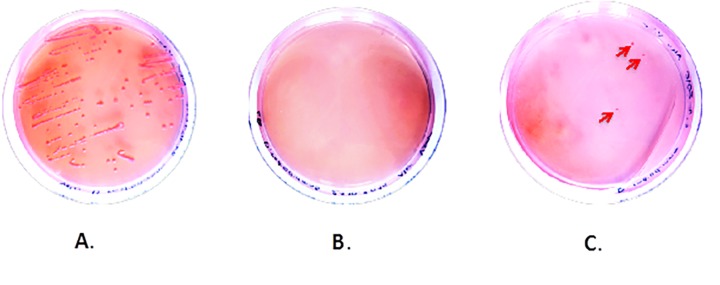
Visible colonies on seeded Petri dishes (TGY medium base) after 10 days of growth **A.** Positive Control (many colonies); **B.** Negative Control (no colonies);** C.** Test sample (3 pink colonies).

**Table 2** summarizes the number of pink colonies observed on Petri dishes after seeding with incubated material. In both experiments positive controls gave growth of pink colonies, with no such colonies on the dishes with negative controls. At the same time, multiple pink colonies were observed in some test Petri dishes, as would be expected in case of self-assembly (**Figure 2**).

In the first experiment, growth was observed for liposomes mixed with RNAs (Test 1) as well as for liposomes mixed with RNAs, DNAs and proteins (Test 4). In total, 10 colonies were observed in 5 test Petri dishes.

In the second experiment, growth was observed on Petri dishes with Test 2 (liposomes with DNAs) and Test 4. In total, 9 colonies were observed in 5 test Petri dishes.

In total, 19 colonies were observed on 10 test plates in both experiments, while zero growth was observed on 27 control plates.

**Table 2:** Number of Petri dishes (plates) with pink bacterial colonies in two experiments for corresponding solutions

	Test 1 RNA in liposomes	Test 2 DNA in liposomes	Test 3 proteins in liposomes	Test 4 RNA + DNA + proteins in liposomes	Negative control: M9 in liposomes	Negative control: M9 with DNA, RNA, proteins without liposomes	Positive control: M9 with DNA, RNA, proteins in liposomes and D. Radiodurans
Exp. 1 Number of plates with colonies growth/out of the total	4/9	0/9	0/9	1/9	0/9	0/9	9/9
Exp. 1 Number of colonies per plate with colony growth	1,2,3,3	0/9	0/9	1	0/9	0/9	many
Exp. 2 Number of plates with colonies growth/out of the total	0/9	3/9	0/9	2/9	0/9	0/9	9/9
Exp. 2 Number of colonies per plate with colony growth	0	1,2,2	0	1,3	0	0	many

**Figure 3:**
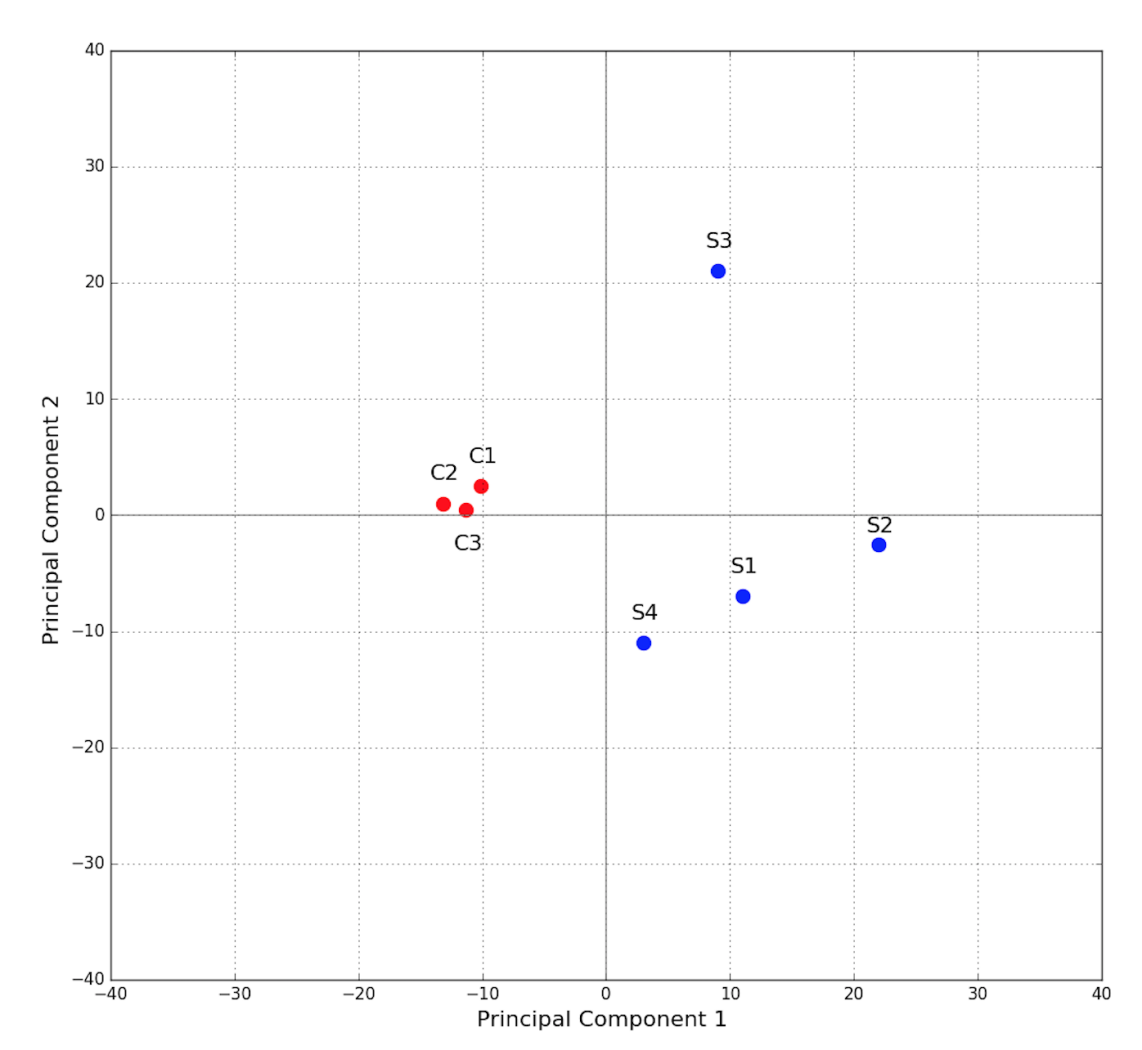
Principal component analysis of the proteome data from the 1st experiment C – positive controls; S – test samples: S1, S2 and S3 are obtained from the colonies on a Petri dish containing Test 1 (liposomes with RNAs), while S4 was obtained from Test 4 (liposomes with RNAs, DNAs and proteins).

In the proteomics analysis, 1031 proteins from *Deinococcus radiodurans *were identified and quantified in the test and control samples with at least 2 unique peptides per protein. The analysis confirmed that all test samples contained mostly proteins from *Deinococcus radiodurans*, even through some *E. coli* proteins were also detected (probable carry-over from previous proteomics experiments on the same equipment). Spread of the sample points in a plot produced by principal component analysis (PCA) revealed that all control samples had identical proteomes, while the proteomics signatures of the test samples were more diverse in terms of protein abundances (**Figure 3**).

## CONCLUSION

The multiple colonies observed on test plates in two indepepdent experiments, with zero growth on control plates, provide overwhelming evidence in favor of the tested hypothesis. Thus, our simple and straightforward experiments confirmed that *Deinococcus radiodurans *bacteria are capable of self-assembly to a viable state from a dead mixture of biopolymers encapsulated in lyposomes. It was reasonable that the proteomes of the resuscitated bacteria deviated somewhat from that of the original bacteria in terms of protein abundances; yet the protein sequences in the emerged species were undoubtedly of *Deinococcus radiodurans.*

In both experiments, fully reconstituted mixtures containing lipids, nucleic acids and proteins produced upon incubation bacterial growth, while in the absence of either lipids or nucleic acids no growth was observed. The colonies formed in lipids+RNAs (first experiment) and lipids+DNAs (second experiment) were probably due to the presence of small quantities of other biopolymers, e.g., complementary nucleic acids (i.e., DNA in RNA isolation, and vice versa) because of imperfect isolation. The lipids+RNAs and lipids+DNAs were initially considered negative controls, and marked as such (Supplementary Figure 2). It is also likely that the presence of small molecules (e.g., ATP) in these mixtures was vitally important for restarting life processes.

For the success of the experiment, we found the following steps to be critical. First, isolation of RNA, DNA and protein fractions needs to be done very carefully, in order to minimize destruction of labile biopolymers. Also important is the liposome preparation – this has to be done by slow, careful rotation of the vial with the temperature below 40 °C to form thin, homogenous and transparent lipid film.

The results of our study lent strong support to the liposome theory of life^6^. Confinement of biomolecules within a lipid vesicle indeed seems to be the prerequisite for life (re)starting from a dead mixture of biomolecules, as many theories have predicted.

The questions remaining unanswered include those on the exact state from which the viable bacteria emerged. How large and how complex were the nanocells that gave rise to bacteria that eventually formed the observed colonies? What is the minimum time frame needed for self-assembly at given conditions? What changes in composition, function and longevity of the newly formed colonies arise after the reassembling process? Further research is needed to address these issues. Another question worth contemplating is the following. If dead bacteria can self-assemble into a living cell, what consequences can this finding have for the important area of food preservation by pasteurization or radiation treatment?

## KEY POINTS

- **Deinococcus Radiodurans bacteria known for exceptional self-repair properties were found to self-assemble from a mixture of nucleic acids and proteins isolated from lysed bacteria**.
- **Critical for self-assembly was found to be the presence of liposomes encapsulating the nucleic acids and proteins**.
- **The likely scenario involves formation from the mixture of nanocells, multiplication of which eventually gives rise to viable Deinococcus bacteria;**
- **The finding offers an explanation of how first life originated from the dead mixture of biomolecules**.
- **These findings could also explain how canned food can go bad after decades-long storage without visible can corrosion. **
