# Supplementary material for: Self-assembly of Deinococcus radiodurans supports nanocell scenario of life origin
Source: Discoveries (Craiova). 2017 May 8;5(1):e72. doi: 10.15190/d.2017.2 (PMC6941539; doi:10.15190/d.2017.2)
Supplement: Supplementary file 1 [file discoveries-05-072-s001.pdf]

DISCOVERIES 2017, 5(1): e72
DOI: 10.15190/d.2017.2

Supplementary Information

Self-assembly of Deinococcus radiodurans supports nanocell scenario of life origin

Alexandra Bernadotte[1, 3], *Valeriya Semenova*[1], *Vitor A. M. Musial*[1], *Anna Kasprzykowska*[1], *and Roman A. Zubarev*[1,2,*]

[1]Division of Physiological Chemistry I, Department of Medical Biochemistry and Biophysics, Karolinska Institutet, Stockholm, Sweden; [2]SciLifeLab, Stockholm, Sweden; [3]Faculty of Mechanics and Mathematics, Lomonosov Moscow State University, Moscow, 119991, Russian Federation;

* *Corresponding author*: Roman A. Zubarev, PhD, Department of Medical Biochemistry and Biophysics, Karolinska Institutet, Scheeles väg 2, SE-17177 Stockholm, Sweden; E-mail: roman.zubarev@ki.se; Phone: +46 8 524 87594;

Citation: Bernadotte A, Semenova V, Musial VAM, Kasprzykowska A, Zubarev RA. Self-assembly of Deinococcus radiodurans supports nanocell scenario of life origin. *Discoveries* 2017, 5(1): e72. DOI: 10.15190/d.2017.2

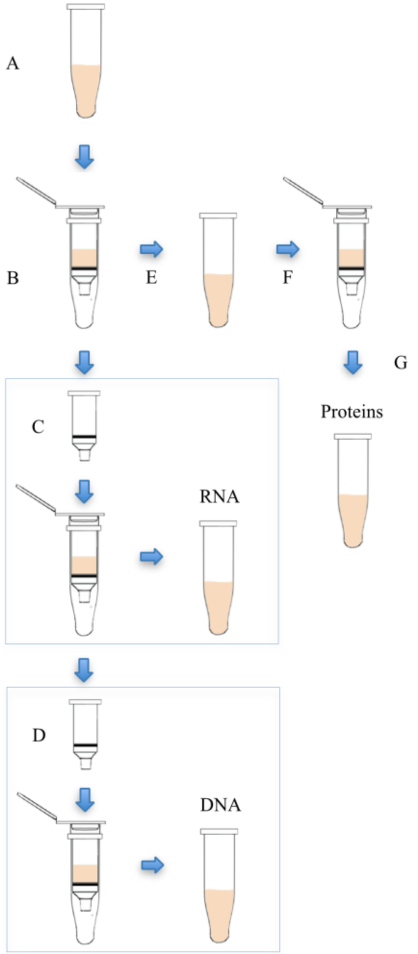

Supplementary Figure 1. Flowchart for purification of RNA, DNA and proteins from a single sample using Norgen's RNA/DNA/Protein Purification Kit.

A. Lysis using Lyse solution. **B.** Binding RNA and DNA to a column. Centrifugation. **C.** Washing with RNA washing buffer and elution with RNA elution buffer; centrifugation. **D.** Washing with DNA washing buffer and elution by DNA elution buffer; centrifugation. **E.** Adjusting the pH of the flowthrough with proteins. **F.** Binding proteins to an activated column. **G.** Washing and elution of proteins.

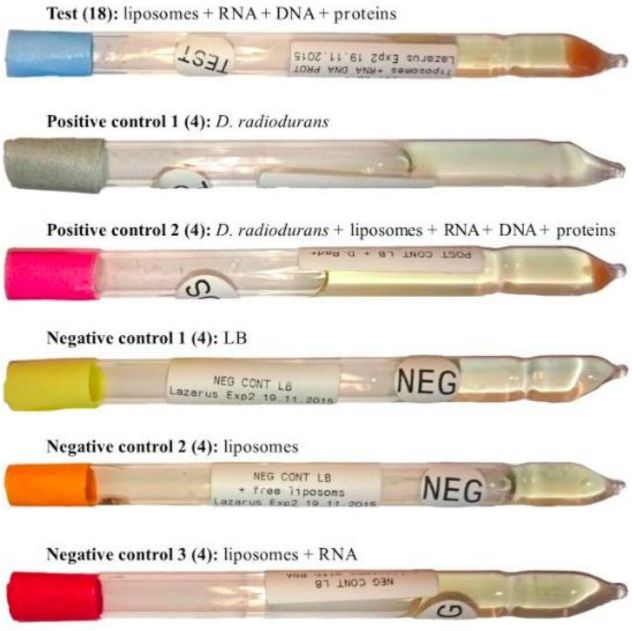

Supplementary Figure 2. **Sealed glass tubes with liposomes and/or RNA, DNA, and proteins suspended in M9 media (Table 1).**
The tubes were both labeled and color-marked to reduce the risk of mix-up, and kept at 4 ^{0}C for 3 weeks before opening and seeding on Petri dishes.

DISCOVERIES is a high quality peer-reviewed, open access, online, multidisciplinary and integrative journal, publishing high impact and innovative manuscripts from all areas related to MEDICINE, BIOLOGY and CHEMISTRY; © 2017, Applied Systems.
